# Xylan, Xylooligosaccharides, and Aromatic Structures With Antioxidant Activity Released by Xylanase Treatment of Alkaline-Sulfite–Pretreated Sugarcane Bagasse

**DOI:** 10.3389/fbioe.2022.940712

**Published:** 2022-07-11

**Authors:** Verônica Távilla F. Silva, Uirajá C. M. Ruschoni, André Ferraz, Adriane M. F. Milagres

**Affiliations:** Departamento de Biotecnologia, Escola de Engenharia de Lorena, Universidade de São Paulo, São Paulo, Brazil

**Keywords:** alkaline-sulfite pretreatment, xylooligosaccharides, xylanase, biorefinery, sugarcane, antioxidants

## Abstract

Xylanase enzymes are useful to fractionate plant biomass, producing xylan, xylooligosaccharides (XOS), and antioxidant-derived XOS. In a biorefinery, pretreated biomass can be digested with xylanase prior to cellulose saccharification, enhancing the product portfolio in the process. With this vision, this study highlighted a wide range of new products attainable from alkaline-sulfite–pretreated sugarcane bagasse by treatments with endo-xylanase under controlled conditions. The developed process provided a crude extract corresponding to 29.7% (w/w) of pretreated sugarcane bagasse. The crude extract included a relatively polymeric glucuronoarabinoxylan fraction, DP2-DP6 xylooligosaccharides, and aromatic compounds. The enzymatically produced extract was fractionated with increasing ethanol concentrations [up to 90% (v/v)], providing precipitation of varied polymeric xylan fractions (48% (w/w) of the crude extract) with average molar masses ranging from 28 kDa to 3.6 kDa. The fraction soluble in 90% ethanol was subjected to adsorption on 4% (w/v) activated charcoal and eluted with an ethanol gradient from 10% to 70% (v/v), thus providing xylooligosaccharides and aromatic fractions. Most of the xylooligosaccharides (74% of the eluted sugars) were washed out in 10%–30% ethanol. DP2 and DP3 structures predominated in the 10% ethanol fraction, while DP5 structures were significantly enriched in the 30% ethanol fraction. Higher ethanol concentrations desorbed xylooligosaccharides associated with higher amounts of aromatic compounds. Total aromatics, phenolic structures, and *p*-hydroxycinnamates predominated in the fractions desorbed with 60% and 70% ethanol. The antioxidant activity of produced fractions correlated with their phenolic contents. Compiled results indicate that a wide variety of products can be prepared from pretreated biomass using xylanase-aided extraction procedures. Recovered fractions presented different features and specific application prospects. Beyond polymeric xylan with low lignin contamination, xylooligosaccharides or even lignin-carbohydrate complexes with antioxidant activity can be included in the biorefinery portfolio based on the currently developed fractionation studies.

## Introduction

Sugarcane bagasse consists primarily of structural polymers such as cellulose, glucuronoarabinoxylan (GAX), and lignin, which give strength to the plant cell wall. Sugarcane bagasse is currently used to generate steam and electricity in mills producing sugar and ethanol in a design that includes electricity sold to the grid (Junqueira et al., 2017; Mendes et al., 2017). Assorted biorefinery schemes indicate more profitable and sustainable processes when part of the sugarcane bagasse is used to add new products to the biorefinery portfolio (Klein et al., 2019). Considering that GAX accounts for 20–30% of the sugarcane bagasse, the use of this fraction would play a key role in upcoming biorefineries if GAX is transformed into high-added value materials (Carvalheiro et al., 2008; Haq et al., 2021).

Sugarcane bagasse processing by alkaline-sulfite treatment generates a highly digestible solid residue enriched in cellulose and GAX (Mendes et al., 2013; Laurito-Friend et al., 2015). The residual GAX has a high degree of arabinose substitution (Ara/Xyl ∼ 0.12) (Sporck et al., 2017). Ferulic acid decreases after alkaline-sulfite pretreatment of sugarcane bagasse, although a small fraction remains ester-linked to arabinose crosslinking to lignin. Moreover ferulic acid, a fraction of *p*-coumaric acid also remains in alkaline-sulfite pretreated sugarcane bagasse, mostly ester-linked to lignin (Reinoso et al., 2018).

GAX removal from pretreated biomass has been shown to significantly improve the subsequent cellulose saccharification step (Hu et al., 2011; Sporck et al., 2017). Thus, extraction of xylans from sugarcane bagasse and other lignocellulosic materials may be desirable for the production of second-generation biofuels because marketable polysaccharides and oligosaccharides can be produced in the biorefinery context (Jayapal et al., 2013; Khaleghipour et al., 2021).

Through an enzymatic approach, GAX removal aided by GH11 xylanases has been used to provide xylans and XOS from alkaline-sulfite pretreated sugarcane bagasse (Sporck et al., 2017; Santos et al., 2019). Xylobiose (X_2_), xylotriose (X_3_), and arabinose-substituted xylooligosaccharides (XOS) are the major products of GH11 xylanases' action on arabinoxylans (Valls et al., 2016). Feruloyl substituents on arabinose do not impede the action of xylanases, and feruloylated XOS may be released in the presence of GH11 xylanase (Faulds et al., 2006; Biely et al., 2016).

Xylanase-solubilized GAX presents high heterogeneity and polydispersity, as well as small amounts of residual lignin contamination (Sporck et al., 2017). Fractions of GAX and XOS with different degrees of polymerization and substitution can be recovered after enzymatic extraction (Cornetti et al., 2020). Fractionation of these materials based on size and/or composition is recommended for certain applications (Van Craeyveld et al., 2008; Hakala et al., 2013; Cornetti et al., 2020). Some fractionation procedures enable the development of a multi-stream integrated biorefinery based on multiple xylan streams to make varied value-added products (Naidu et al., 2018). Separation of high molar mass xylan is mostly carried out by precipitation with alcoholic solutions (Swennen et al., 2005). Such xylans are useful to be incorporated onto eucalyptus fibers for increasing fiber-fiber bonding and yield (Muguet et al., 2011; Cornetti et al., 2020) as well as to prepare polymeric films (Ninõ-Medina et al., 2010; Xiang et al., 2020). The oligomeric and monomeric forms are substrates for platform chemicals (Saini et al., 2022).

The compatibility of xylans and XOS carrying antioxidant activity with cosmetic matrices, food products, and food packing materials has led to a great interest in using these biomass-derived compounds in new commercial products (Amorim et al., 2019; Freitas et al., 2019). For example, XOS with DP up to 10 present prebiotic properties (Van Craeyveld et al., 2008). Feruloylated XOS behaves as phenolic antioxidants due to the high reactivity of the vicinal hydroxyl-methoxy substitution on the aromatic ring (Mathew et al., 2015). XOS fractions, produced from pretreated sugarcane bagasse assisted by the *Clostridium thermocellum* xylanase, presented antioxidant activity which was dependent on XOS concentration and the presence of phenolic compounds (Mandelli et al., 2014). Concentration-dependent antioxidant activity was also described for sugarcane bagasse-derived XOS obtained by enzymatic hydrolysis (Bian et al., 2013). XOS lacking ferulic acid ramifications also presented some antioxidant properties (Valls et al., 2018; Bouiche et al., 2020). In these cases, acidic XOS fractions showed higher antioxidant activity than neutral XOS, probably associated with phenolic contaminants occurring in the acidic fraction. Despite the increasing interest in the antioxidant properties of XOS, the antioxidant activity of its derived fractions has not been comprehensively described up to date, probably because complex mixtures of XOS have been evaluated in the *in vitro* antioxidant assays.

According to the desired application, XOS can be purified by different methods, such as ion exchange resin, membrane separation, and activated charcoal adsorption (Zhang et al., 2018). In this context, the current study used endo-xylanases under controlled conditions to prepare a crude enzymatic extract from alkaline-sulfite pretreated sugarcane bagasse that contains GAX, XOS, and aromatic compounds. Fractionation of this extract by GAX precipitation with increasing ethanol concentrations and subsequent use of activated charcoal for adsorption/desorption of the soluble xylooligosaccharides provided a wide range of products in which the antioxidant activity was closely dependent on the phenolic contents of each fraction.

## Material and Methods

### Chemicals and Enzymes

Chemicals were purchased from Sigma-Aldrich (St. Louis, MO, United States). Commercial GH11 xylanase (luminase PB-200) was donated by Verenium. Trolox (6-hydroxy-2,5,7,8-tetramethylchroman-2-carboxylic acid) was used as an antioxidant reference. DPPH (2,2-diphenyl 1-picrylhydrazyl) and ABTS (2,2-azino-bis(3-ethylbenzothiazoline-6-sulfonate) were used as radical sources in antioxidant activity assays.

### Pretreated Sugarcane Bagasse Preparation

The pretreatment of sugarcane bagasse consisted of an alkaline-sulfite step using 5% NaOH and 10% sodium sulfite, at 120°C for 2 h, as described previously (Sporck et al., 2017). The solid yield of the prepared alkaline-sulfite pretreated material was 75.8%, corroborating previous work (Reinoso et al., 2018). The chemical composition of the original sugarcane bagasse and the resulting pretreated material was determined according to standard protocol (Ferraz et al., 2000). The uronic acid content was determined as anhydro-uronic acid content by the carbazole-sulfuric acid method, as previously described by Brienzo et al. (2009). Glucuronic acid was used as a calibration standard for this method. Ferulic and *p*-coumaric acids were determined after severe alkaline treatment (4 mol/L NaOH at 170°C for 3 h), as previously described (Masarin et al., 2011). All determinations were performed in triplicate, and average values followed by standard deviations are reported in the text. A summary of the chemical composition of the materials used in the current work is shown in Table 1.

**TABLE 1:** Chemical composition (g/100 g, dry matter) of the original and alkaline-sulfite–pretreated sugarcane bagasse used in the current experiments.

Component	Sugarcane bagasse	Alkaline-sulfite–pretreated bagasse
Glucan	40.4 ± 0.2	49.8 ± 0.2
Xylan	22.4 ± 0.3	24.4 ± 0.2
Arabinosyl	2.6 ± 0.1	2.8 ± 0.1
Acetyl groups	3.3 ± 0.1	0.3 ± 0.1
Uronic acid	2.5 ± 0.2	2.1 ± 0.3
Ferulic acid	1.5 ± 0.1	0.7 ± 0.1
Total lignin	21.1 ± 0.4	13.2 ± 0.4
*p*-coumaric acid	3.7 ± 0.4	1.9 ± 0.4
Extractives	4.5 ± 0.5	2.0 ± 0.2
Solid yield (%)	100	75.8

### Enzymatic Treatment of Alkaline-Sulfite–Pretreated Sugarcane Bagasse

Xylan and xylooligosaccharide extraction from alkaline-sulfite pretreated bagasse were initially assessed under varied reaction conditions. Pretreated sugarcane bagasse (50 mg) was incubated with 1 ml of xylanase solution prepared in phosphate buffer at pH 6.0 or 8.0 inside 5 ml microcentrifuge tubes. The xylanase activity was determined according to Bailey et al. (1992) using birchwood xylan as the substrate. The enzyme activity was measured at each defined pH to adjust the same enzyme activity in each experiment, which was fixed at 5 U/g substrate. Reaction temperatures were set at 50, 60, or 70°C. The mixtures were agitated at 120 cycles per min with sampling after 3, 6, and 24 h of reaction. At the end of each reaction period, the samples were boiled for 5 min and centrifuged (4,750 g for 20 min). Reactions were performed in triplicate, and average extraction yield values followed by standard deviations are reported in the text.

Experiments were scaled up to 10 g of pretreated sugarcane bagasse and 200 ml of enzyme solution in 1-L Erlenmeyer flasks. Initial reaction pH was adjusted to 6 with HCl instead of using buffering to facilitate subsequent product analysis and purification. The reaction was conducted at 60°C and 120 cycles per min for 24 h. The reacted material was boiled for 10 min to inactivate enzymes, and centrifuged at 4,600 g for 5 min.

In both reaction protocols, the supernatants were submitted to dilute acid post-hydrolysis (4% w/w H_2_SO_4_ at 121°C for 1 h) according to Templeton et al. (2010) and analyzed for xylose and arabinose contents by HPLC (Sporck et al., 2017).

### Fractionation of the Enzymatic Hydrolysate

Enzymatically produced supernatants prepared at the scaled-up protocols were fractionated into four fractions by graded ethanol precipitation. In the first step, ethanol was added under continuous stirring to a final concentration of 30% (v/v). The mixture containing enzymatically released materials and ethanol solution was stored at 4°C for 16 h. Precipitated material was recovered as a pellet after centrifugation at 4,600 g for 30 min at 4°C. Afterward, the ethanol concentration was further increased to 60% (v/v) in the previous supernatant. The new precipitated fraction was recovered as described earlier. The procedure was repeated once again to reach 90% (v/v) ethanol in the supernatant. The pellets recovered in each step were lyophilized, and a portion of each pellet (10 mg) was dissolved in 10 ml of Milli-Q water and analyzed by HPSEC and HPLC.

The supernatant of the final mixture containing 90% (v/v) ethanol was adsorbed onto activated charcoal prepared from coconut shell (particle size 1–2 mm, surface area 120 m^2^ g^−1^, Labsynth Ltda, Brazil). For this, 50 ml of the supernatant was mixed with 2 g of activated charcoal and shaken at 80 rpm for 18 h. The solids were recovered by filtration and washed with aqueous ethanol solutions to desorb oligosaccharide fractions. The washing process started with 25 ml of 10% (v/v) ethanol aqueous solution. The remaining solids were further washed out with 25 ml ethanol aqueous solutions with ethanol concentrations increasing up to 70% (v/v). The solvent of each desorbed fraction was partially evaporated under reduced pressure when the final recovered volume was recorded. All produced fractions were characterized by UV-Vis spectroscopy for molar mass distribution, xylooligomers, total sugars, total phenolics, and antioxidant activity, as described as follows. The ratio of recovered fraction volume/initial volume was considered for component concentrations and antioxidant activity calculations.

### Characterization of the Glucuronoarabinoxylans and Oligosaccharide Fractions

Molecular mass distribution of samples was determined by gel filtration using Biogel P-30 columns (50 cm × 1 cm ID; Bio-Rad, Hercules, CA, United States) for GAX and Biogel P-2 (100 cm × 1.6 cm ID; Bio-Rad, Hercules, CA, United States) for xylooligosaccharides. The columns have been equilibrated with NaOH at pH 10 and eluted at 0.3 ml/min with the same eluent. The eluted fractions (3 ml) were assayed by the phenol-sulfuric method to detect total eluted sugars (Dubois et al., 1956). The system was calibrated with xylose and dextran standards (Pharmacosmos) in the 1,000–40,000 molecular mass range. The column systems based on Biogel P-30 and Biogel P-2 provided linear calibration curves with R^2^ values of 0.9969 and 0.9887, respectively.

The concentration of xylooligosaccharides (DP_2_ to DP_6_) was determined by HPLC using a Waters HPLC system equipped with two Bio-Rad columns in series in this sequence, HPX-87C and HPX-42A (Bio-Rad, Hercules, CA, United States) at 65°C, coupled with a refractive index detector at 35°C. Deionized water was the mobile phase at 0.5 ml/min. Xylooligosaccharides were also analyzed by thin-layer chromatography (TLC). Aliquots of 2 μl were applied on silica gel-coated aluminum sheets (Merck). TLC plates were developed in a solvent system composed of ethyl acetate/acetic acid/2-propanol/formic acid/water 25:10:5:1; 15 (v/v). The sugars were visualized by spraying the TLC plates with 1-naphthyl-ethylenediamine dihydrochloride reagent (1.8 g in 200 ml methanol and 20 ml concentrated sulfuric acid) followed by heating at 100°C for 5 min (Bounias, 1980).

The phenol-sulfuric acid method was used to determine the total concentration of carbohydrates present in GAX samples recovered in fractionation experiments. A calibration curve was prepared using xylose as a standard (Dubois et al., 1956).

The content of total phenolics was assessed by the Folin–Ciocalteu assay (Athukorala et al., 2006). The reaction consisted of 100 µl distilled water, 20 µl ethanol (95% v/v), 20 µl sample, and 10 µl Folin–Ciocalteu reagent (50% v/v). After standing for 5 min, 20 µl sodium carbonate (5% w/v) was mixed, and the mixture was kept in the dark for 1 h. The absorbance of the reaction product was measured at 725 nm. The reaction reference was prepared by replacing the sample with distilled water. A calibration curve was prepared using gallic acid as a phenolic standard.

UV spectra were recorded from enzymatic hydrolysate and its fractions in an Evolution-201 spectrophotometer (Thermo Scientific, United States).

The antioxidant capacity of the biorefined fractions was evaluated with two different *in vitro* methods using Trolox as a reference antioxidant. In the ABTS radical scavenging assay, ABTS radical was initially prepared by mixing 2.5 ml of 7 mmol/L ABTS with 44 µl of 140 mmol/L potassium persulfate for 16 h in the darkness at 25°C. The prepared solution was diluted in water until the absorbance at 730 nm was 0.70 ± 0.05. Then, 180 µl of the diluted ABTS cation radical solution was mixed with 20 µl of sample. The loss of color of blue-green ABTS radical cation was measured after 10 min at 730 nm in a microplate reader (Valls et al., 2018).

The DPPH radical scavenging assay used 50 µl of 0.1 mM freshly prepared DPPH solution (prepared in ethanol 95% v/v) mixed with 50 µl of sample. The mixture reacted for 120 min in the darkness at 25°C. After this period, the absorbance was measured at 517 nm in a microplate reader (Bian et al., 2013).

## Results

### Enzymatic Treatment of Alkaline-Sulfite–Pretreated Sugarcane Bagasse for Glucuronoarabinoxylan and Xylooligosaccharide Extraction

Alkaline-sulfite–pretreated sugarcane bagasse was the substrate for a set of enzymatic treatments designed to recover methylglucuronoarabinoxylan (GAX) and xylooligosaccharides. The chemical composition of the pretreated sugarcane bagasse included 49.8% glucan, 29.6% GAX, and 13.2% lignin (Table 1). Removal of 52% of the original lignin from sugarcane bagasse during pretreatment assured high digestibility to the pretreated substrate (Laurito-Friend et al., 2015). Pretreated material retained most of the glucan and 82% of the original GAX, resulting in a polysaccharide-enriched substrate prepared from sugarcane bagasse. Intense deacetylation and partial deferuloylation of GAX occurred during pretreatment (93% and 65%, respectively) owing to the alkaline pretreatment conditions. In contrast, almost half of the original *p-*coumaric acid remained in the pretreated bagasse mainly linked to residual lignin, corroborating previous work (Reinoso et al., 2018).

The high digestibility of the alkaline-pretreated sugarcane bagasse conferred by 52% lignin removal and almost complete GAX deacetylation during pretreatment (Laurito-Friend et al., 2015; Santos et al., 2019) open the opportunity for recovering GAX and xylooligosaccharides from such materials through enzymatic processing (Bian et al., 2013; Hakala et al., 2013). Tailoring enzyme types and dosages, as well as reaction conditions in these processes, help to provide diversified products in biorefinery (Biely et al., 2016; Freitas et al., 2019). In the current work, varied polymeric GAX and xylooligosaccharides with antioxidant activity were targeted in the biorefinement procedures applied to fractionate pretreated sugarcane bagasse.

**FIGURE 1:**
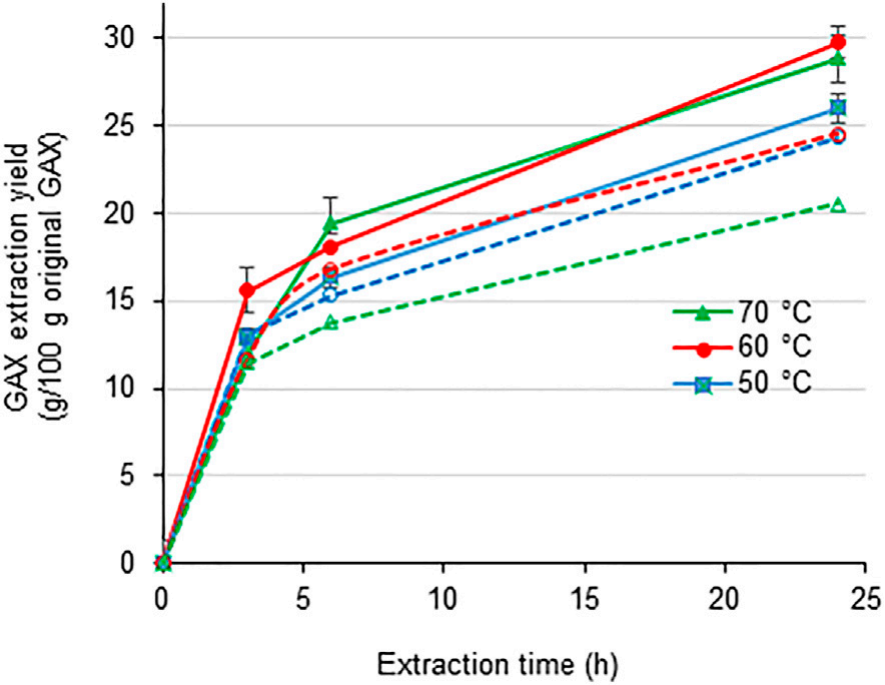
Total yields of glucuronoarabinoxylan (GAX) extracted from alkaline-sulfite–pretreated sugarcane bagasse mediated by commercial xylanase (luminase at 5 U/g substrate) acting at different reaction pH values and temperatures. pH 6.0 (filled symbols connected by continuous lines); pH 8 (empty symbols connected by dotted lines).

A set of reaction conditions was screened to reach high recovery yields of GAX and xylooligosaccharides. The reaction temperature and pH affected total GAX extraction yields mediated by luminase action on alkaline-sulfite pretreated bagasse (Figure 1). Total GAX extraction yields increased progressively with reaction time, but extraction rates diminished significantly after 8 h of reaction. The highest total GAX extraction yield occurred at pH 6, reaching almost 30% of the GAX contained in pretreated bagasse after 24 h of reaction at either 60°C or 70°C. However, the extraction efficiency slowed at 50°C. At higher pH (pH 8), the total GAX extraction yield decreased to 24.5% at 50–60°C and even more (20.5%) at the highest reaction temperature (70°C). These data are in agreement with studies indicating that luminase displayed the best enzyme stability at pH 6, retaining 100% activity after 150 min. Decreasing stability was observed at pH 7 and pH 8 (Santos et al., 2019).

The Ara/Xyl ratio of GAX released at pH 6.0 and 60°C (0.15) was slightly higher than that detected in the sugarcane pretreated substrate (0.11—Table 1), suggesting that extracted GAX is enriched in decorations, probably because this characteristic increases GAX solubility (Gomes et al., 2015).

### Fractionation of Extracted Glucuronoarabinoxylans and Xylooligosaccharides

Extraction of pretreated sugarcane bagasse with luminase was scaled-up at pH 6 and 60°C to provide a crude GAX extract subjected to subsequent fractionation. This extract was then fractionated by precipitation under increasingly ethanol concentrations. During precipitation with 30% (v/v) ethanol, a high molar mass GAX fraction (28 kDa) became insoluble, representing 6% of the originally extracted GAX (Table 2). Increasing ethanol concentrations to 60% (v/v) and 90% (v/v) provided the other two fractions representing 11% and 31% of the total GAX originally extracted, respectively. The last two GAX fractions presented progressively lower Mw values than the fraction precipitated at 30% (v/v) ethanol. However, a significant fraction (52%) remained in solution even at 90% (v/v) ethanol owing to the predominance of short xylooligosaccharides in the extract, whose molar mass distribution indicated an Mw value as low as 1 kDa. Precipitated and 90% (v/v) soluble samples also varied according to the total phenolic contents. The sample with the highest Mw value also contained high amounts of phenolic compounds, which decreased significantly in the fractions precipitated at higher ethanol concentrations. These data suggest an enrichment in contaminant lignin or lignin-carbohydrate complexes in the sample with the highest Mw value, whereas the fractions precipitated at higher ethanol concentrations are relatively free of phenolic structures. The fraction soluble at 90% (v/v) ethanol also contained significant amounts of phenolic compounds (Table 2).

**TABLE 2:** Fractionation yield and molar mass distribution of enzymatically extracted glucuronoarabinoxylan (GAX) from alkaline-sulfite–pretreated sugarcane bagasse after precipitation by increasing ethanol concentrations.

Fraction	Fraction yield (%)^a^	Mw (kDa)	Mn (kDa)	Mw/Mn	Phenolics (mg/g)^c^
Precipitated with 30% ethanol	6	28.1	25.6	1.1	83.4 ± 2.2
Precipitated with 60% ethanol	11	9.4	8.5	1.0	10.6 ± 0.3
Precipitated with 90% ethanol	31	3.6	2.8	1.3	3.6 ± 0.1
Soluble at 90% ethanol	52^b^	1.0	0.8	1.3	150.5 ± 14.1

a Expressed as the weight percentage of total extracted GAX.

b Determined by the difference between the originally extracted GAX and the precipitated fractions.

c Phenolics expressed as mg of gallic acid per g total sugars.

**FIGURE 2:**
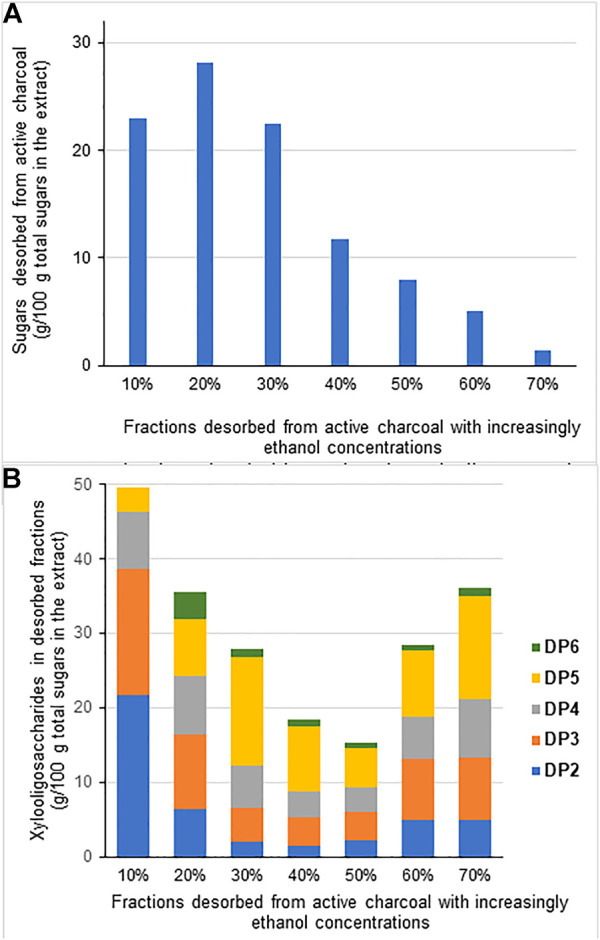
Fractionation of the GAX extract that remained soluble in 90% (v/v) ethanol, as described in Table 2. Soluble material was adsorbed in active charcoal and desorbed by increasing ethanol concentrations. **(A)** Sugars desorbed in each fraction related to the total sugars in solution; **(B)** xylooligosaccharides with DP2 to DP6 identified in the same fractions illustrated in **(A)**.

**FIGURE 3:**
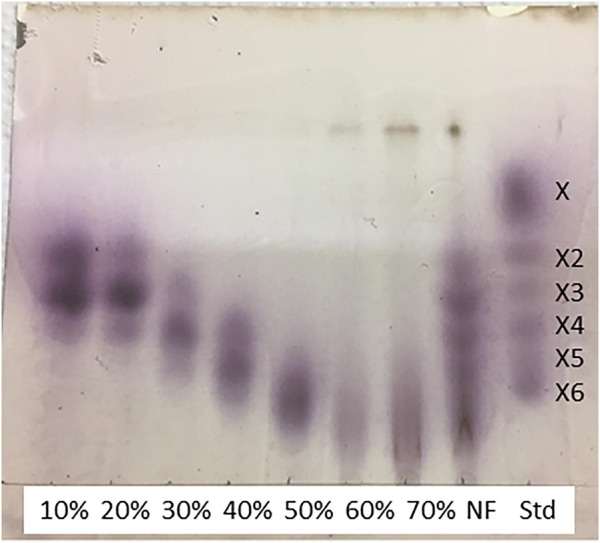
Thin-layer chromatography elution of xylooligosaccharides contained in the GAX extract that remained soluble in 90% (v/v) ethanol, as described in Table 2. Soluble material was adsorbed in active charcoal and desorbed by increasing ethanol concentrations. Each fraction is identified by the ethanol concentration used in the desorption step. **NF** is the non-fractionated original sample; **Std** refers to xylooligosaccharide standards eluted in the same run.

The fraction that did not precipitate in 90% (v/v) ethanol was further fractionated by adsorption in active charcoal and selective desorption by ethanol solutions with incremental concentrations (Figure 2). Figure 2A shows the yield of each desorbed carbohydrate fraction, indicating that most of the carbohydrates were desorbed by 10%–30% (v/v) ethanol. Carbohydrates detected in each desorbed fraction corresponded to DP2-DP6 structures (Figure 2B). DP2 and DP3 structures were mostly released at 10% and 20% (v/v) ethanol, and the fraction desorbed with 30% (v/v) ethanol was attractive to recover DP5 structures once it presented a high XOS yield (Figure 2A) and contained 50% of all available DP5 from the fractionated material (Figure 2B). DP5 XOS also predominates in the fraction extracted by 70% ethanol (Figure 2B), but this fraction represented a low total XOS yield (Figure 2A). Further analysis of the XOS by TLC evidenced the presence of additional structures with DP higher than 6, which were desorbed at 50–70% (v/v) ethanol concentrations (Figure 3). TCL spots observed on top of 60% ethanol, 70% ethanol, and NF rows, not assigned to XOS, are probable lignin fragments not linked to XOS.

**FIGURE 4:**
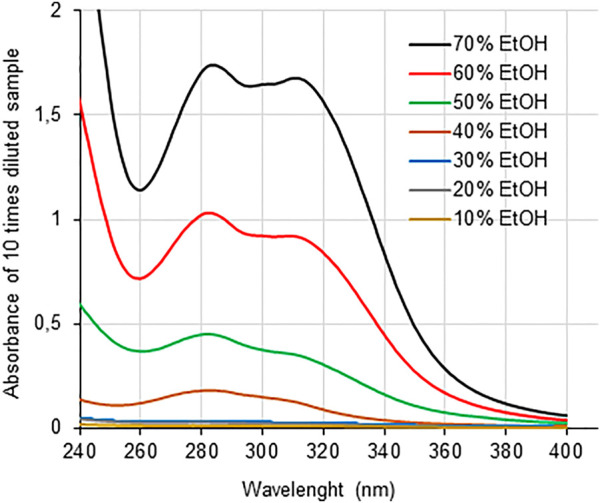
UV spectra of GAX-derived fractions from the extract that remained soluble in 90% (v/v) ethanol, as described in Table 2. Soluble material was adsorbed in active charcoal and desorbed by increasing ethanol concentrations. Each fraction is identified by the ethanol concentration used in the desorption step.

UV spectra of the fractions desorbed from active charcoal indicate that all samples contain aromatic moieties derived from contaminant lignin or lignin-carbohydrate complexes as well as *p*-hydroxycinnamates (Figure 4). This is supported by clearly defined bands at 280 nm and 315 nm, corresponding, respectively, to oxygen-substituted aromatic moieties and to α-β unsaturations conjugated with aromatic rings, typical of *p*-coumarate and ferulate esterified into grass cell wall components (He and Terashima, 1991). The UV absorption intensity markedly increased from samples desorbed from low to high ethanol concentrations (Figure 4), contrasting with the total carbohydrate contents that followed an inverse behavior (Figure 2A). Total phenolic contents in these fractions varied from zero to 565 μM expressed as gallic acid, following a similar behavior of UV absorption intensity (Table 3). These data indicate that the fractions desorbed with ethanol concentrations from 40% (v/v) contained progressively more phenolic structures and fewer carbohydrates, which is consistent with the higher solubility of phenolic compounds at higher ethanol concentrations (Sun et al., 2015; Goldman et al., 2019).

**TABLE 3:** Phenolic contents in GAX-derived fractions from the extract that remained soluble in 90% (v/v) ethanol, as described in Table 2. Soluble material was adsorbed in active charcoal and desorbed by increasing ethanol concentrations.

Ethanol concentration used to desorb GAX-derived fractions from active charcoal (%)	Total phenolic contents expressed as gallic acid (μM)
10	0.0
20	20.6 ± 0.6
30	18.2 ± 1.8
40	83.6 ± 4.7
50	274.7 ± 0.6
60	533.6 ± 15.2
70	563.9 ± 23.3

### Antioxidant Activity of Biorefined Extracts

**FIGURE 5:**
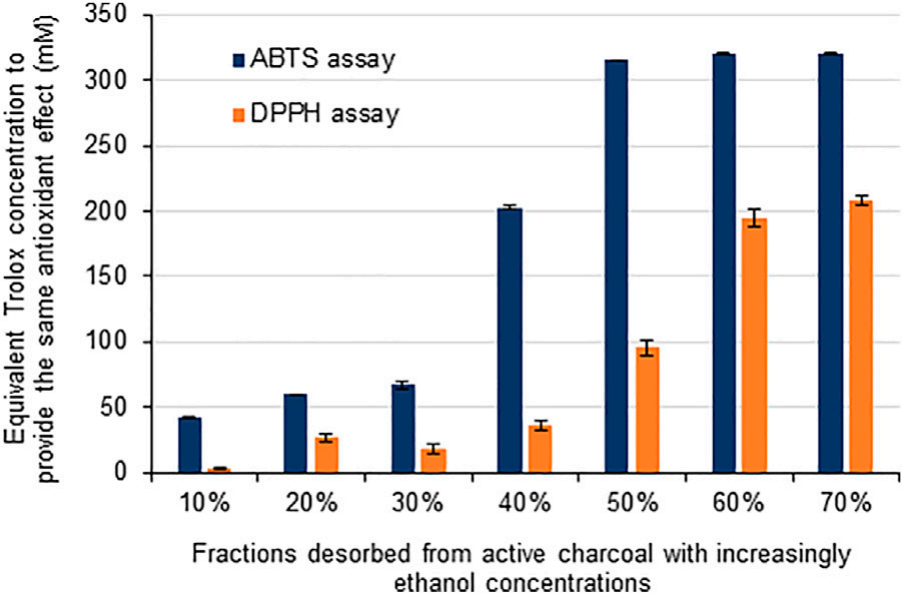
Antioxidant activity of xylooligosaccharide fractions described in Table 3.

Two different *in vitro* assays assessed the antioxidant activity of the currently evaluated fractions. Both methods (DPPH and ABTS) involve the capacity of the antioxidants to act as radical scavengers in the solution. ABTS radical is first produced by ABTS oxidation by persulfate (Valls et al., 2018) whereas DPPH is a crystalline stable radical providing a colored solution when dissolved in 95% ethanol (Bian et al., 2013). Antioxidants disproportionate these radicals providing color loss of the assayed solutions, which is measured at defined wavelengths (Melo-Silveira et al., 2012; Shahidi and Zhong, 2015). Trolox is a reference antioxidant usually used for comparison purposes (Bian et al., 2013). In the ABTS method, Trolox concentrations up to 300 µM provided a linear correlation with ABTS radical discoloration whereas over 320 μM were enough to completely disproportionate radicals after 10 min of reaction. In contrast, the color decrease of DPPH in solution was less pronounced in the presence of Trolox, with maximal discoloration restricted to 36% of the initial absorption values for Trolox concentrations over 300 μM. In the ABTS method, the fractions desorbed from active charcoal from 50% (v/v) ethanol concentrations performed similarly to 320 µM Trolox (Figure 5), providing complete radical disproportionation after 10 min of reaction. For the DPPH assay, the equivalent Trolox concentrations detected in the desorbed fractions were lower owing to the lowest sensitivity of the method (Martysiak-Żurowska and Wenta, 2012).

**FIGURE 6:**
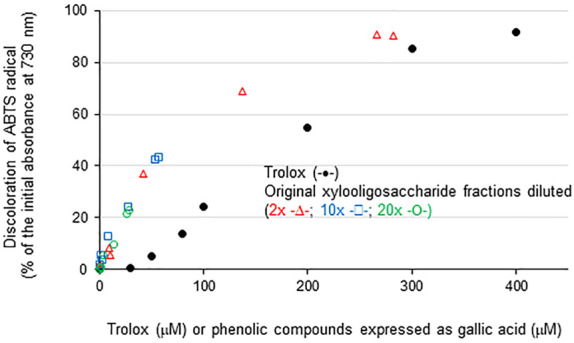
Correlation of total phenolic contents detected in xylooligosaccharide fractions desorbed from active charcoal by increasing ethanol concentrations and their antioxidant activity revealed by ABTS radical scavenging assays. Trolox at varied concentrations is shown in black-filled symbols. Varied phenolic contents of the xylooligosaccharide fractions were obtained by progressive dilutions of the fractions listed in Table 3.

Phenolic contents in the fractions desorbed with 60% and 70% ethanol exceeded the equivalent Trolox concentration necessary for complete radical disproportionation in the ABTS method (Table 3), motivating progressive dilution of the samples for a study of antioxidant efficiency compared to Trolox. Diluted samples presented antioxidant activity correlated with their phenolic contents and higher activity than Trolox at phenolic concentrations up to 270 μM expressed as gallic acid (Figure 6). The origin of the antioxidant activity detected in the studied fractions is probably associated with the occurrence of assorted compounds contained in each fraction that include ferulate linked to XOS, *p*-coumarate linked to lignin fragments, and lignin phenolics contaminating the samples (Figure 4). Pure phenolic compounds evaluated in similar assays indicate that ferulic acid, which contains vicinal hydroxyl and methoxyl groups in the aromatic ring, presents higher antioxidant activity than *p*-coumaric acid which lacks the methoxyl group (Baderschneider and Winterhalter, 2001). Feruloyl and *p*-coumaryl groups attached to short oligosaccharides have shown higher antioxidant activity than lignin derivatives (Tung et al., 2009; Malunga and Beta, 2015; Valls et al., 2018). However, complex mixtures of aromatic moieties occurring in the currently evaluated samples limited further detailed evaluation of their structure versus antioxidant performance.

## Discussion

**FIGURE 7:**
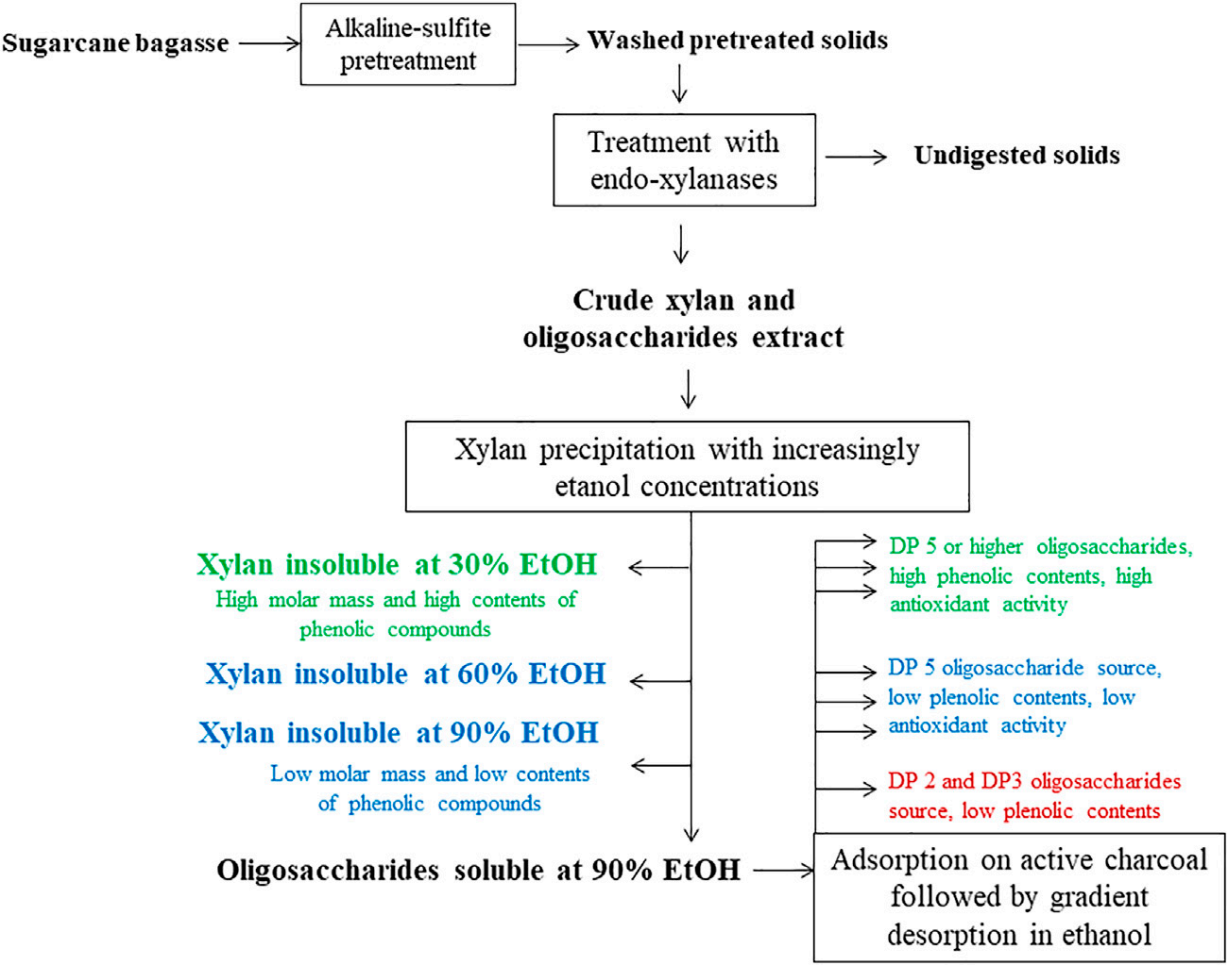
Sugarcane bagasse biorefinery scheme focused on developing new products from the GAX fraction retained after alkaline-sulfite pretreatment.

Although sugarcane bagasse has a current important role in energy co-generation in sugar and ethanol mills, several studies related to sugarcane bagasse lignocellulose biorefinement indicate that adding new products to the biorefinery portfolio is critical to improving process sustainability and profitability (Yamakawa et al., 2018; Rosales-Calderon and Arantes, 2019). In this context, new studies regarding sugarcane bagasse byproducts with potential biochemical activity are useful to point out new biorefinery goods with high commercial interest. In the present work, we developed a new biorefinement scheme to recover diversified xylan-derived products from alkaline-sulfite pretreated sugarcane bagasse (Figure 7). The alkaline-sulfite process contrasts with other pretreatments that cause major xylan degradation (Carvalheiro et al., 2008; Chaturvedi and Verma, 2013). During alkaline-sulfite pretreatment, lignin was the major removed component whereas most of the glucan and GAX were preserved in pretreated solids. Despite being retained at high yields, GAX suffered extensive deacetylation and partial deferuloylation, which turned it especially suitable for xylanase-aided extraction. The use of commercial xylanases at low loads (5 U/g substrate) under optimal reaction conditions permitted the recovery of almost 30% of the xylan contained in pretreated sugarcane bagasse. Moreover, using graded ethanol concentrations to fractionate the originally prepared extract permitted the production of GAX fractions with varied characteristics according to their molecular size and levels of aggregated phenolic compounds. For example, there are strong pieces of evidence indicating that xylan adsorption in pulp fibers is closely dependent on the average molar mass and molar mass distribution (Cornetti et al., 2020). Providing three different ranges of molar mass and varied contents of phenolic components, the current biorefinement scheme seems suitable to provide interesting GAX fractions for use as fillers in papermaking (Petzold-Welcke et al., 2014; Xiang et al., 2020) and food packing paper materials (Martínez-Abad et al., 2015; Mikkonen et al., 2015).

The fraction of the extracted GAX that did not precipitate even at 90% (v/v) ethanol was further adsorbed in active charcoal and desorbed by graded ethanol concentrations to yield seven xylooligosaccharide fractions. Ethanol concentrations of 10%–20% (v/v) provided fractions enriched in DP2-DP3 xylooligosaccharides and low phenolic contents. Ethanol-derived desorption at 30% (v/v) provided a DP5 xylooligosaccharide-enriched fraction also presenting low phenolic contents, suggesting that fractions produced from desorption with low ethanol concentrations are suitable for further recovery of pure xylooligosaccharides. Ethanol concentrations from 40% (v/v) interestingly desorbed xylooligosaccharides containing high phenolic contents, which conferred high antioxidant activity to these fractions (Rao and Muralikrishna, 2006; Ratanasumarn and Chitpraser, 2020; Mohamed et al., 2021). Compared to Trolox, the oligosaccharide fractions desorbed from 50% ethanol concentration provided similar antioxidant activities to 320 µM Trolox. Progressive dilution of the xylooligosaccharide fractions indicated that the antioxidant activity was closely dependent on the phenolic content of the fractions and that concentrations up to 270 μM (expressed as gallic acid equivalents) presented higher antioxidant activity compared to the Trolox reference. Therefore, the xylooligosaccharides obtained by desorption from 50% (v/v) ethanol seemed suitable for food products and cosmetic preparations owing to their high antioxidant activity (Bian et al., 2013; Almendinger et al., 2020; Chen et al., 2021).

The overall developed process seems suitable to diversify products in a biorefinery processing sugarcane bagasse. Associating alkaline-sulfite pretreatment, xylanase-aided extraction of GAX and xylooligosaccharides with the currently developed fractionation of the crude GAX-extract provided some new GAX-derived products, including polymeric GAX with molar masses ranging from 28 to 3.6 kDa and seven xylooligosaccharide fractions, including DP5-enriched fractions and selected fractions with high antioxidant activity, mostly associated with their high phenolic contents. Previous work demonstrated that GAX extraction also benefits subsequent saccharification of remaining solids by commercial enzyme cocktails once the residual polysaccharides became more accessible to cellulolytic enzymes (Sporck et al., 2017). After completing this biorefinery schedule, residual lignin from the final saccharification step can be converted into marketable lignosulfonates, as recently reported in Heinz et al. (2022).

## Conclusion

Digestion of alkaline-sulfite–pretreated sugarcane bagasse by endo-xylanase under controlled conditions highlighted that a wide range of new products with varied molecular sizes and levels of aggregated phenolic compounds can be produced in the sugarcane bagasse biorefinery context. Most of the xylooligosaccharides recovered from the hydrolysate had DP2 to DP5 and low phenolic contents, turning them more suitable for use as prebiotics. High phenolic contents and the UV-spectroscopic characteristics of the xylooligosaccharide fractions produced by desorption from 50% (v/v) ethanol concentrations suggest that these fractions contain *p*-hydroxycinnamates and lignin or lignin-carbohydrate complexes. The occurrence of high antioxidant activity in the same fractions corroborates a series of reports demonstrating high antioxidant activity of similar plant biomass-derived components. Data considered altogether support the use of the currently developed biorefinement scheme to add new products in a sugarcane bagasse biorefinery with great potential for improved profitability and sustainability.

## Data Availability

The raw data supporting the conclusion of this article will be made available by the authors, without undue reservation.
